# Deep Circumflex Iliac Artery Osteoseptocutaneous Flap as a Reconstruction Method for Distal Radius Recurrent Giant Cell Tumour in the Case of a Bilateral Peroneal Magna Artery: An Eight-Year Follow-Up

**DOI:** 10.7759/cureus.69547

**Published:** 2024-09-16

**Authors:** Nor Hazla Mohamed Haflah, Patrina Chan Ke Jing, Levin Kesu Belani, Mohamed H Sani, Arman Zaharil Mat Saad, Faisham Wan Ismail

**Affiliations:** 1 Orthopaedics, Faculty of Medicine, Unversiti Kebangsaan Malaysia, Kuala Lumpur, MYS; 2 Orthopaedics and Traumatology, Faculty of Medicine, Universiti Kebangsaan Malaysia, Kuala Lumpur, MYS; 3 Plastic and Reconstructive Surgery, Management and Science University Medical Centre, Shah Alam, MYS; 4 Orthopaedics, Prince Court Medical Centre, Kuala Lumpur, MYS

**Keywords:** autografts, deep circumflex iliac artery, distal radius gct, free vascularised fibular flap, giant cell tumour of bone, long-term outcome, variant arterial anatomy, wrist arthrodesis

## Abstract

A vascularized fibula flap is an option to reconstruct osseous and soft tissue defects involving distal radius malignancy with massive soft tissue involvement. This reconstruction method is a strong anatomical construct for wrist arthrodesis and flexible septocutaneous tissue for closure. However, in rare cases of bilateral peroneal magna artery, a vascularized fibula flap is not a suitable option given its potential risk of limb ischemia. We report the case of a 35-year-old lady with recurrent distal radius giant cell tumor with bilateral peroneal magna artery, whereby a vascularized fibula flap is not a reconstruction option for the distal radius. In this case, we opted to use the deep circumflex iliac artery (DCIA) flap to reconstruct the defect. This case highlights the importance of clinical assessment and Doppler evaluation before harvesting a vascularized fibula graft and the DCIA flap as an alternative option for reconstruction of the distal radius with a good functional outcome eight years post-operation.

## Introduction

Giant cell tumor (GCT) of bone is a benign but locally aggressive tumor that represents 5% of all primary bone tumors [1]. It has a peak incidence between 20 and 50 years of age and is more prevalent in females [1]. The tumor typically arises from the metaphyseal and epiphyseal ends of a long bone, commonly occurring at the distal femur, proximal tibia, and distal end of the radius [1]. The goal of the treatment is to achieve satisfactory removal of the tumor, reduce the chance of recurrence, and preserve limb function. Despite its benign histopathology nature, GCT has a high rate of local recurrence of 20.5%, commonly seen 28.8 months after surgery depending on the initial treatment [2]. The recurrence rate is higher in those who underwent intralesional curettage alone or with soft tissue extension. A more aggressive approach, such as en bloc resection with reconstruction, has shown a lower recurrence rate at the price of poor functional outcomes. Several reconstruction options exist, such as arthroplasty or arthrodesis with structural bone graft (non-vascularized or free vascularized fibula autograft or allograft), centralization of the distal ulna, and endoprosthesis [3]. In this case, one of the challenges was finding a suitable flap due to massive soft tissue involvement and an incidental finding of an absent posterior tibialis artery and dorsalis artery pulse, which was later diagnosed as a bilateral peroneal magna artery. A deep circumflex iliac artery (DCIA) flap was used as it was able to provide a large concave segment of cancellous bone suitable for reconstruction of the upper extremity. It is a great alternative to a fibula flap when the lower extremities are injured or there is inadequate peroneal artery inflow.

## Case presentation

**Figure 1:**
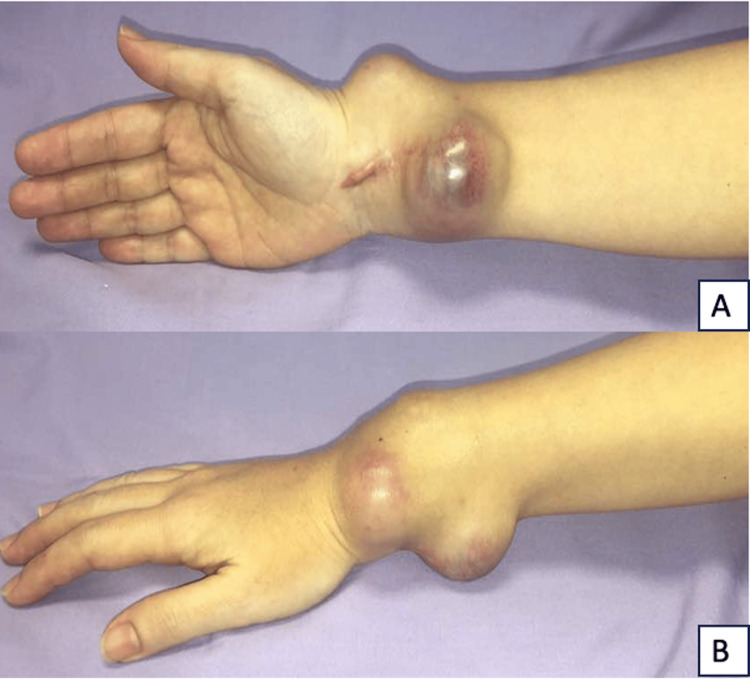
Swelling caused by a giant cell tumor A: Swelling over the volar surface of the right wrist over the previous surgical incision; B: Swelling over the dorso-radial aspect of the right wrist

**Figure 2:**
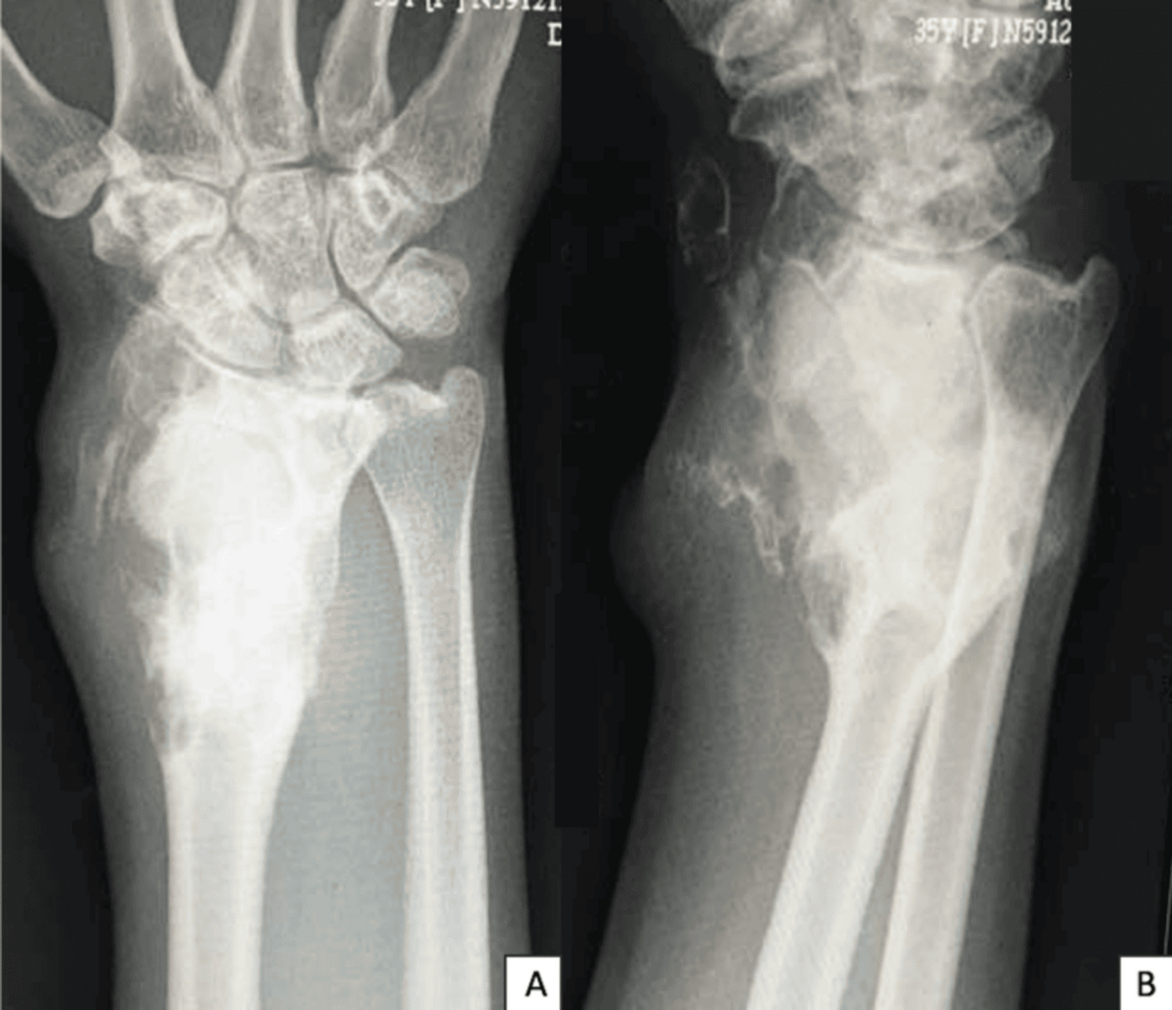
Lytic lesion with cortical breakage at the right distal radius with polymethyl methacrylate in situ seen on the anteroposterior (A) and lateral (B) radiograph of the right wrist

A 35-year-old lady was diagnosed with a giant cell tumor of the right distal radius and presented with recurrent swelling of the right distal radius six months after initial treatment with extended curettage, adjuvant phenol, and polymethyl methacrylate osseous reconstruction. The swelling was gradually increasing in size, and the wrist motion was grossly restricted due to pain. There were two major lobulated swellings over the dorso-radial and volar aspects of the wrist, which were firm and marked with skin infiltration (Figure 1). The hand grip had good power, and the median and ulnar nerve motor and sensory function were preserved. A radiograph showed destruction and bony fragmentation of the radial cortex with soft tissue extension; however, the radiocarpal joint was preserved with polymethyl methacrylate in situ (Figure 2).

**Figure 3:**
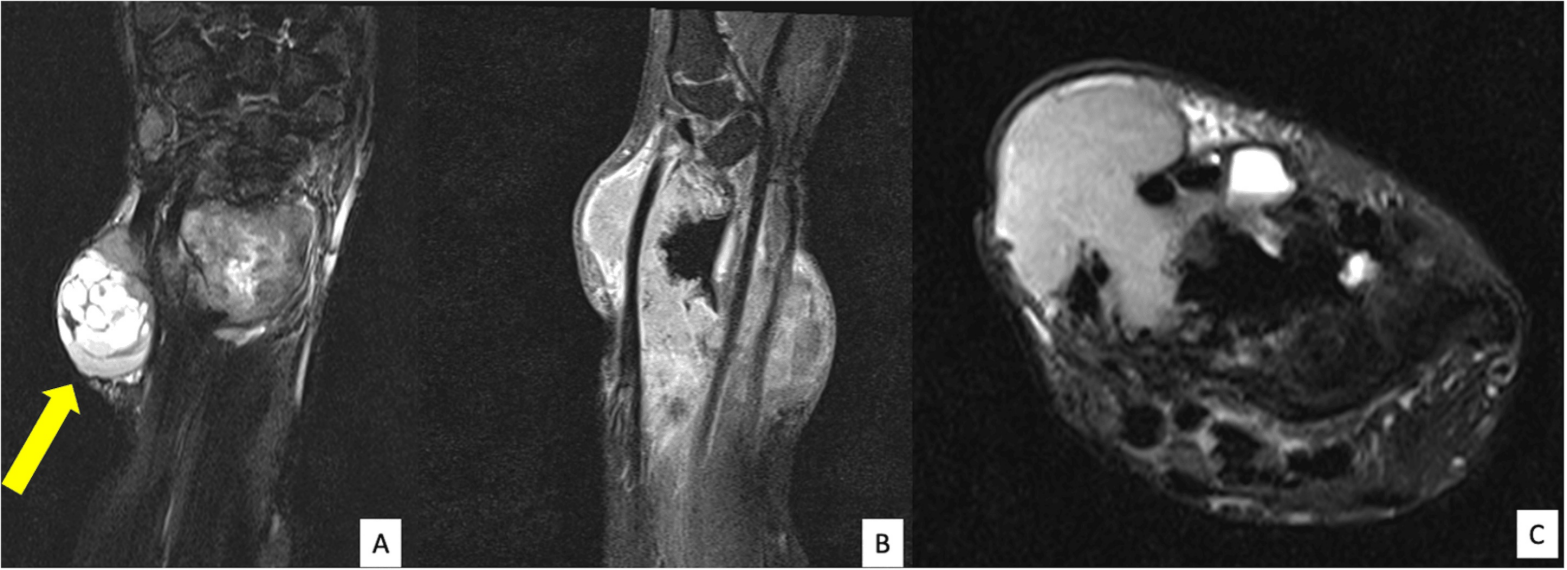
The MRI imaging of the right wrist in coronal (A), sagittal (B), and axial (C) views shows a soft tissue mass measuring 5.0 cm x 4.0 cm (AP x W). A multiloculated cystic lesion is also seen (yellow arrow) measuring 2.6 cm x 3.0 cm x 3.7 cm (AP x W x CC). AP: Anteroposterior, W: Width, CC: Craniocaudal

An MRI for local staging revealed the mass to be a soft tissue component encasing the distal radius until the level of the wrist joint. The mass measured 5.0 cm x 4.0 cm (anteroposterior (AP) x width (W)) and demonstrated similar signal intensity to the intramedullary lesion. This extraosseous soft tissue component extended volarly, pushing the flexor tendons away. No clear plane was demonstrated in between. Dorsally, the mass insinuated between the first and second extensor tendons with encasement of the rest of the extensor tendons. Distally, there was an extension into the wrist joint with minimal high T2 signal intensity within the lunate bone. A new multiloculated cystic lesion was also seen within the subcutaneous tissue of the distal radius measuring 2.6 cm x 3.0 cm x 3.7 cm (AP x W x craniocaudal (CC)). This lesion abutted the underlying tendons and muscles. The visualized radial and ulnar neurovascular bundles were intact, with a clear plane visualized between the vessels and the mass. Extensive skin infiltration at the palmar aspect and dorsoradial aspect confirmed on MRI, warranted resection (Figure 3).

**Figure 4:**
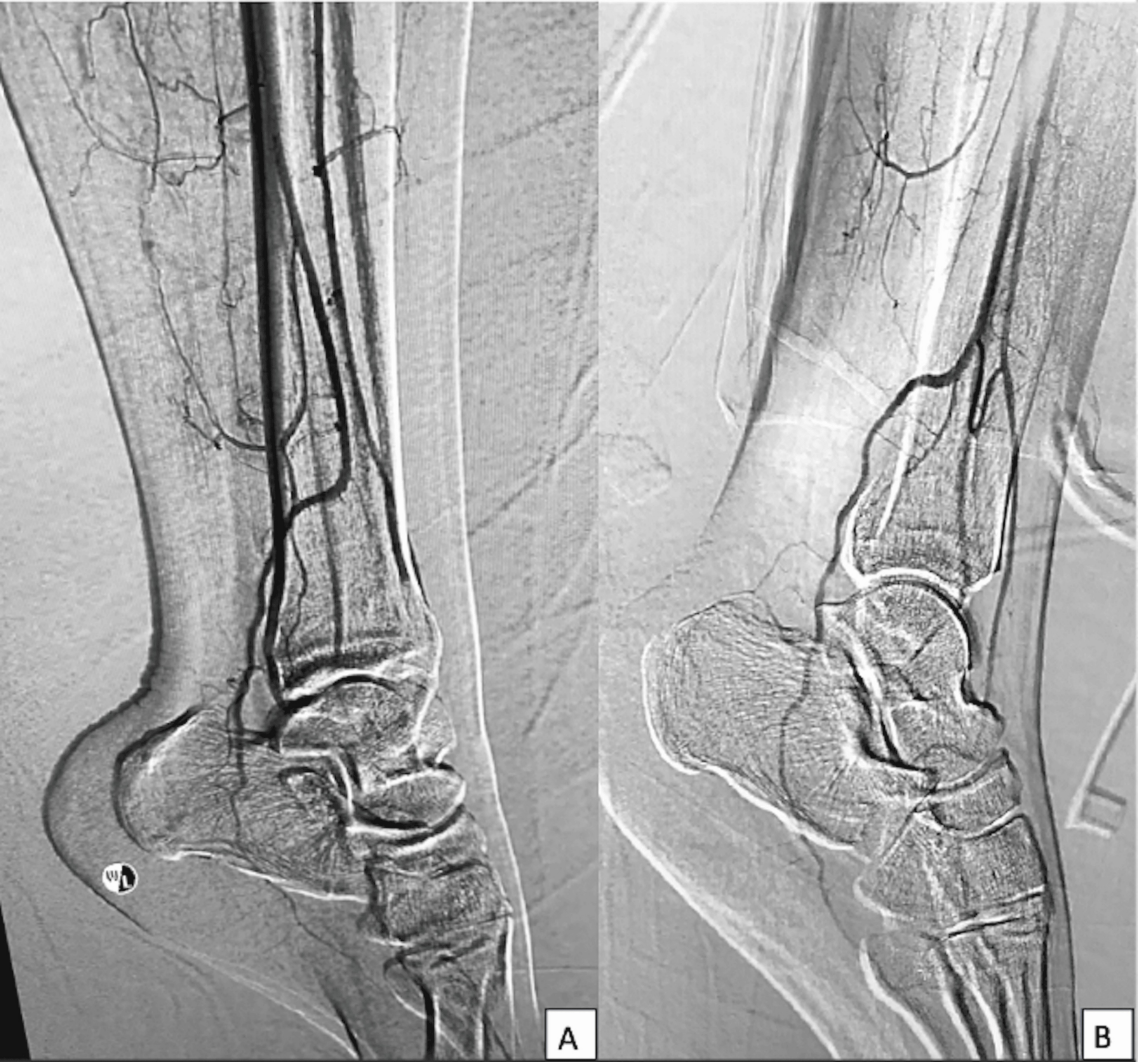
Digital subtraction angiography shows the peroneal artery supplying the posterior aspect of the foot and becoming a lateral plantar artery (A). Also noted is the absence of the posterior tibial artery, and the anterior tibial artery is present up to the dorsalis pedis but is small in caliber (B).

Due to the extensive involvement of soft tissue and skin, the patient was planned for wide local resection and osteocutaneous fibula graft reconstruction. However, upon evaluation of the donor limb, we noted that the bilateral posterior tibialis artery pulse was absent, and the dorsalis pedis artery pulse was weak. These findings were confirmed with a Doppler scan. Lower limb digital subtraction angiography (DSA) confirmed the absence of the posterior tibial artery, small caliber anterior tibial artery, and the peroneal artery as the sole supply to the foot bilaterally, a condition known as 'peroneal magna artery' (Figure 4). 

**Figure 5:**
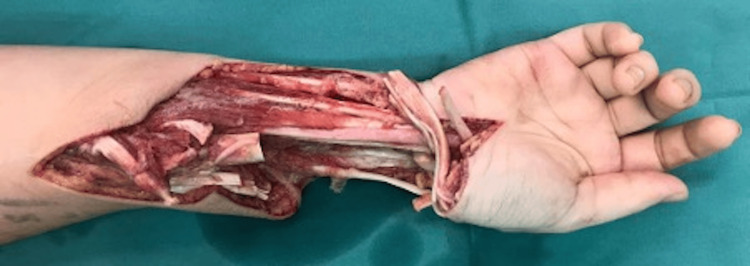
Intraoperative image showing the wide local excision of the tumor with excision of the common flexor and extensor tendons to achieve good margins. The defect on the radius measured approximately 11 cm.

**Figure 6:**
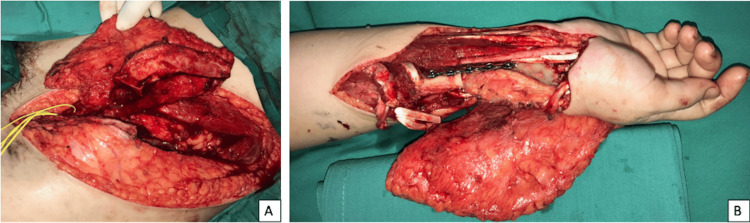
Intraoperative image A: Osteoseptocutaneous iliac bone graft with DCIA vessel harvested from the left ilium; B: The osteoseptocutaneous graft was fixed at the right distal radius and the wrist was fused with a double dynamic compression plate. The DCIA was anastomosed end-to-end with the radial artery. DCIA: Deep circumflex iliac artery

**Figure 7:**
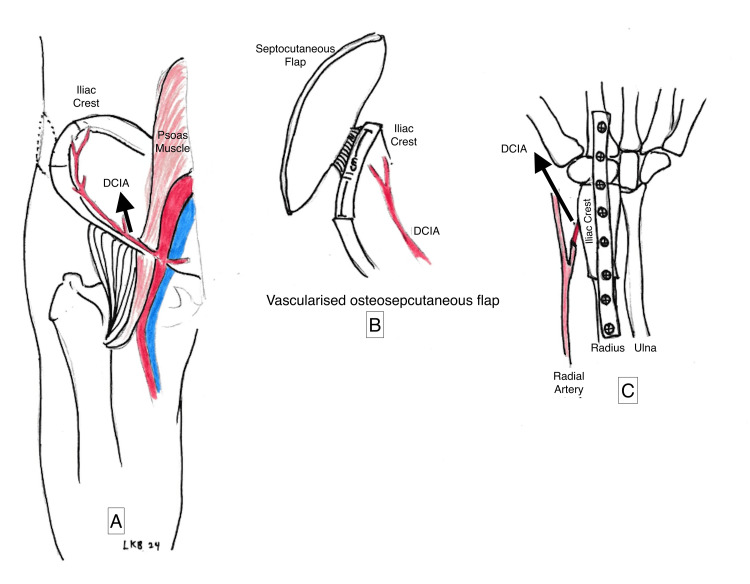
The septocutaneous flap and iliac crest were measured and harvested together with the DCIA (A & B). The osteoseptocutaneous graft was fixed to the distal radius after wide local excision and secured with a dynamic compression plate; the DCIA was anastomosed with the radial artery (C). DCIA: Deep circumflex iliac artery Hand-drawn illustrations created by the authors.

**Figure 8:**
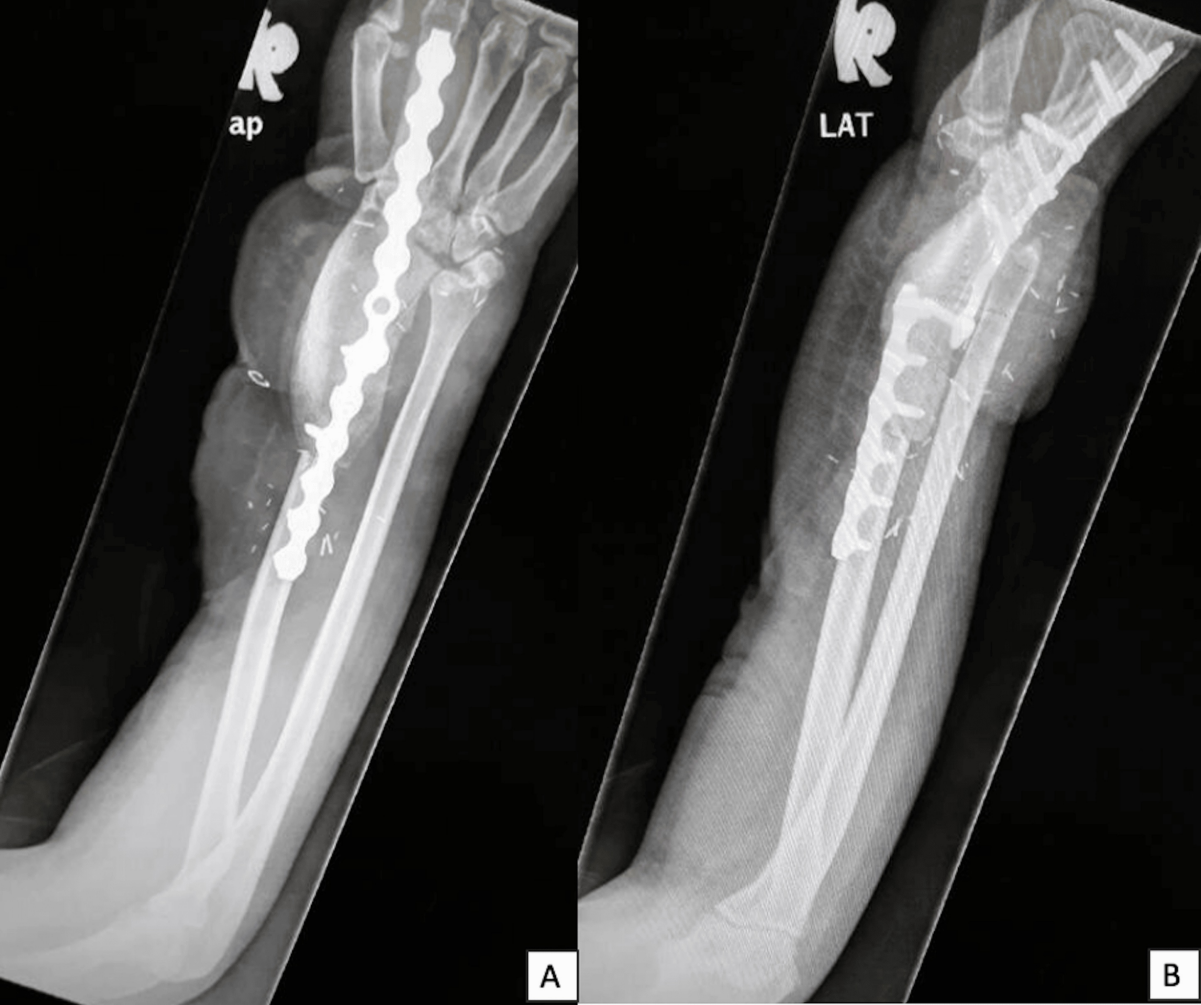
Postoperative radiograph of the reconstruction of the distal radius defect using DCIA osteoseptocutaneous iliac graft and fusion of the right wrist A: Anteroposterior view; B: Lateral view

The recurrent tumor involved the entire distal radius and infiltrated the pronator quadratus and the synovium of the common flexor and skin. Dorsally, the fibro-osseous extensor tunnel limits the extent of recurrence to the extensor tendon and skin. The tumor was excised en-bloc involving 11 cm of the distal radius, the entire pronator quadratus, and the capsule of the wrist joint. The extensor of the thumb and extensor digitorum communis were elevated and freed from its fibro-osseous tunnel. The flexor to the thumb and index finger was cut and freed from its synovium attachment for clearance. The common flexor and extensor tendon of the wrist were cut in segments and included as surgical margins (Figure 5). Proximal row carpectomy was performed to gain good margins. The large distal radius defect was reconstructed with a DCIA osteoseptocutaneous flap harvested from the left iliac bone (Figure 6 A). The osteoseptocutaneous flap was then used to reconstruct the defect of the right distal radius and was secured with two 3.5 reconstruction plates to fuse the wrist joint (Figure 6 B and Figure 7). Fixation was checked using an image intensifier, and the position of the flap and plate was deemed satisfactory (Figure 8). The final histopathology for the recurrent swelling is in keeping with giant cell tumor. 

**Figure 9:**
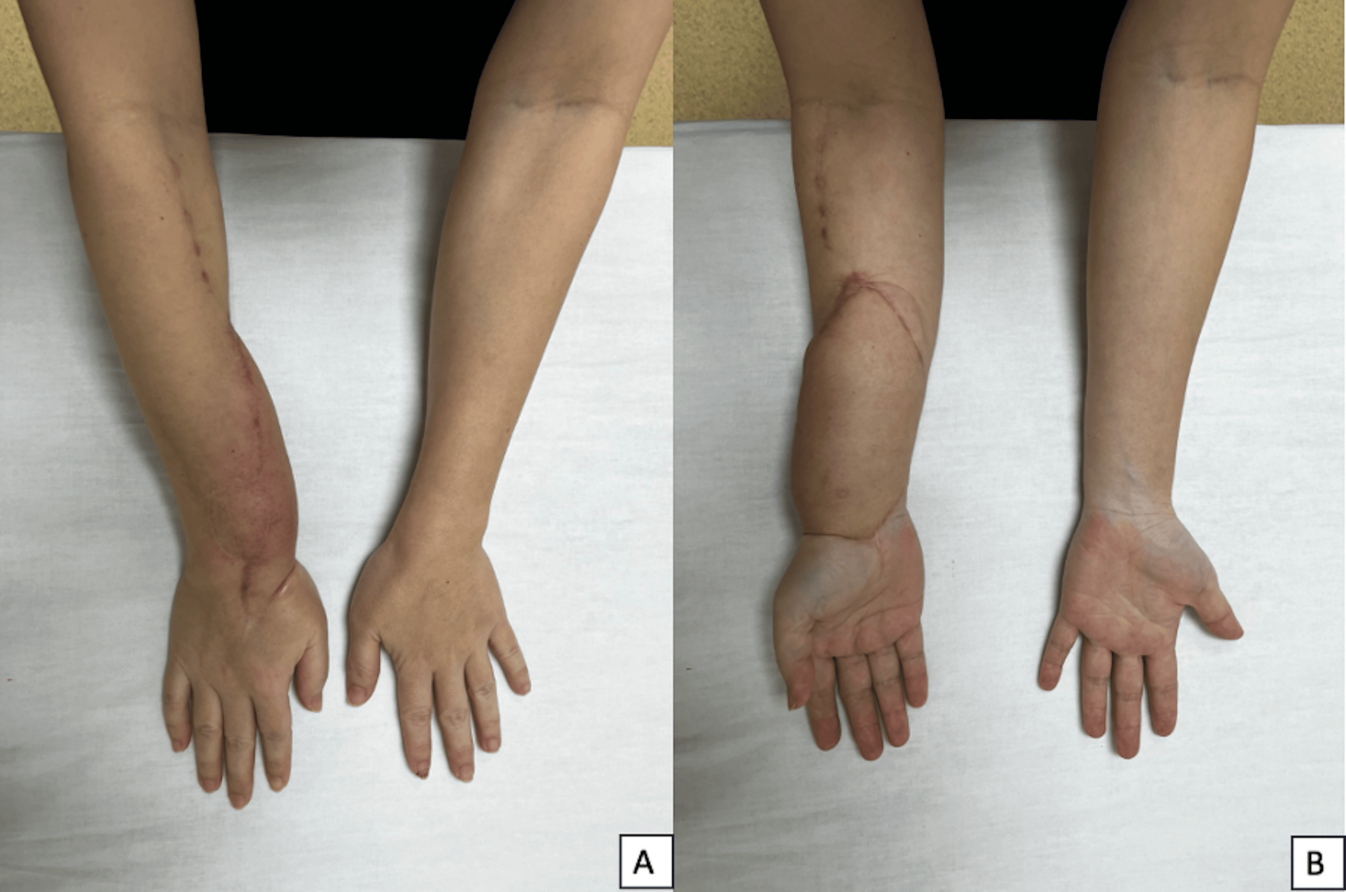
Full pronation (A) and supination (B) of the right forearm compared to the contralateral side

**Figure 10:**
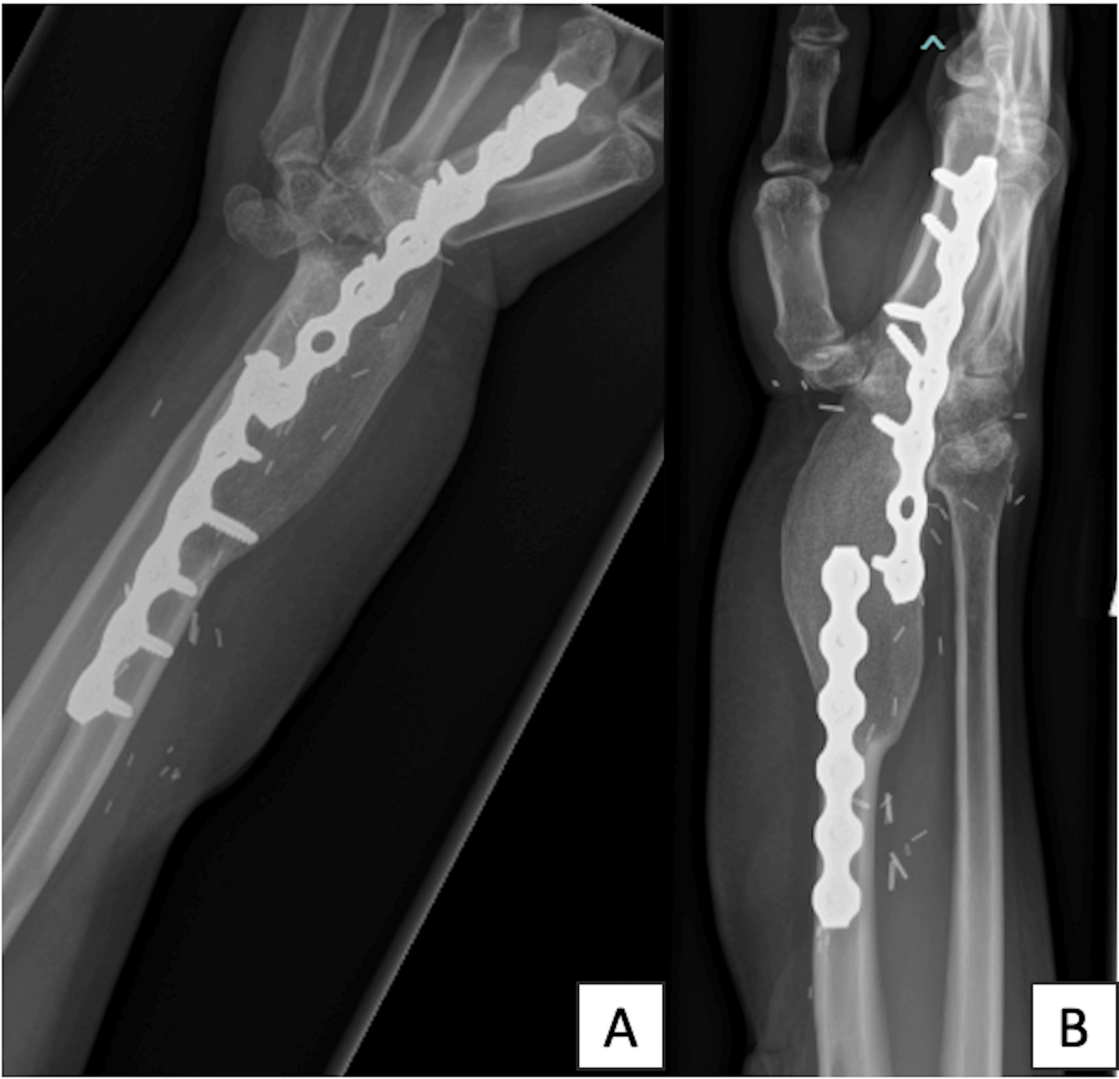
The radiograph taken eight years postoperatively shows good wrist fusion. A: Anteroposterior view; B: Lateral view

Currently, eight years after surgery, the patient's hand function is good with stable wrist joint fusion and good grip. The pronation and supination functions are almost equal compared to the normal site (Figure 9). The latest radiograph shows that the wrist has fused (Figure 10). There is no local recurrent and the lungs are free from metastases. The donor site was painless without complaint.

## Discussion

There are many options for reconstructing the distal radius defect that are both biological and non-biological [3]. The fibula is a long, straight bone that is anatomically similar in diameter and shape to the forearm bones [4]. In cases where the tumor has breached the cortex and there is an extension of soft tissue destroying the proximal carpus, the vascularized fibula graft is a good autograft option [5]. The osteocutaneous vascularized fibular graft provides better soft tissue coverage over the defect site, leading to better tendon gliding and stability of the wrist joint [5]. Although arthrodesis of the wrist limits the range of motion of the wrist joint, a stable wrist fusion in cases of proximal row carpectomy leads to good grip strength, reflecting a better functional outcome [5]. 

Clinical assessment of the vascular status of the lower limb is important prior to deciding on the use of a vascularized fibular graft [6]. The fibula has two blood supplies: the endosteal blood supply and the main supply by the peroneal artery. However, there is a variation in the blood supply of the lower limb where the anterior and posterior tibial arteries may be hypoplastic and the peroneal artery is the main blood supply to the lower limb; a condition known as peroneal magna [7]. This condition can be identified by palpating the posterior tibialis artery pulse and dorsalis pedis artery pulse. In our case, the bilateral posterior tibial artery pulse was absent and the bilateral dorsalis pedis pulse was weak. A suspicion of bilateral peroneal magna was confirmed by a DSA of the bilateral lower limb. Although vein mapping of the lower limb in such circumstances is controversial, we decided to proceed with the DSA to have better preoperative planning as up to 55.2% of a surgical approach is influenced by preoperative MR angiography [8]. Given that this patient has peroneal magna bilaterally, a vascularized fibular flap was no longer an option for reconstruction due to the risk of causing the patient to have ischemia in her lower limb should the fibular be harvested [6]. Hence, the decision to reconstruct the distal radius bone gap with a DCIA iliac bone graft. 

The use of a non-vascularized iliac graft for reconstruction of a short segment distal radius with arthrodesis wrist has been proven to produce a good functional outcome [9-11]. The pronation and supination were near normal as maintenance of the radial height produced anatomical ulnar minus for rotational movement of the wrist. However, the complication rate related to non-union, hardware failure, and the need for additional surgery was up to 30% [9].

Hence the decision to reconstruct the distal radius defect using DCIA osteoseptocutaneous iliac graft to reduce the risk of non-union with arthrodesis of the wrist joint to provide a good long-term clinical outcome. Given the length of the defect, the DCIA iliac bone graft harvested was curved, leading to difficulties in achieving optimal position for wrist arthrodesis, which is in 30 degrees of dorsiflexion and slight ulnar deviation, especially in this case where proximal row carpectomy was performed. However, it has a smooth surface both dorsally and ventrally to allow smooth tendon glide with the septocutaneous flap for better soft tissue reconstruction.

## Conclusions

Our case highlights the importance of clinical assessment and Doppler evaluation before harvesting vascularized fibular grafts. In this case of bilateral peroneal magna, the option of vascularized fibula graft was not suitable, hence the decision to reconstruct the defect using DCIA iliac crest graft was made. Even though the long segment iliac crest is not anatomically straight for the reconstruction of the distal radius, adequate radial length tension produces good stability of the ulna for rotation, which is comparable with the outcome of using a vascularized fibula graft. Arthrodesis of the wrist provided the patient with a good functional outcome after eight years of surgery with no recurrence, metastasis, or morbidity at the donor site.

## References

[1] Jha Y, Chaudhary K (2023). Giant cell tumour of bone: a comprehensive review of pathogenesis, diagnosis, and treatment. Cureus.

[2] Pitsilos C, Givissis P, Papadopoulos P, Chalidis B (2023). Treatment of recurrent giant cell tumor of bones: a systematic review. Cancers (Basel).

[3] Chobpenthai T, Poosiripinyo T, Warakul C (2023). Reconstruction after en bloc resection of a distal radius tumor. An updated and concise review. Orthop Res Rev.

[4] Petrella G, Tosi D, Pantaleoni F, Adani R (2021). Vascularized bone grafts for post-traumatic defects in the upper extremity. Arch Plast Surg.

[5] Choo CY, Mat-Saad AM, Wan-Azman WS, Wan Z, Nor-Azman MZ, Yahaya S, Faisham WI (2018). Functional outcome after treatment of aggressive tumours in the distal radius: comparison between Reconstruction using proximal fibular graft and wrist fusion. Malays Orthop J.

[6] Nadeem W, Ferrell JK, Taylor CB (2024). Peronea magna: an important anatomic variant impacting fibula-free flap reconstruction. OTO Open.

[7] Kim D, Orron DE, Skillman JJ (1989). Surgical significance of popliteal arterial variants. A unified angiographic classification. Ann Surg.

[8] Lohan DG, Tomasian A, Krishnam M, Jonnala P, Blackwell KE, Finn JP (2008). MR angiography of lower extremities at 3 T: presurgical planning of fibular free flap transfer for facial reconstruction. AJR Am J Roentgenol.

[9] Wang T, Chan CM, Yu F, Li Y, Niu X (2017). Does wrist arthrodesis with structural iliac crest bone graft after wide resection of distal radius giant cell tumor result in satisfactory function and local control?. Clin Orthop Relat Res.

[10] Gulia A, Puri A, Prajapati A, Kurisunkal V (2019). Outcomes of short segment distal radius resections and wrist fusion with iliac crest bone grafting for giant cell tumor. J Clin Orthop Trauma.

[11] Kuruoglu D, Rizzo M, Rose PS, Moran SL, Houdek MT (2022). Treatment of giant cell tumors of the distal radius: a long-term patient-reported outcomes study. J Surg Oncol.

